# The mitochondrial genome of the scarlet-backed flowerpecker (*Dicaeum cruentatum* Linnaeus) from southwestern China

**DOI:** 10.1080/23802359.2021.1972484

**Published:** 2021-09-06

**Authors:** Yong Gao, Si Yin, Lei Zhu

**Affiliations:** College of Biological Resource and Food Engineering, Qujing Normal University, Qujing, Yunnan, China

**Keywords:** Scarlet-backed flowerpecker, *Dicaeum cruentatum*, mitogenome, phylogenetic analysis

## Abstract

*Dicaeum* (family Dicaeidae) is a genus of passerines distributed in tropical southern Asia and Australasia. The scarlet-backed flowerpecker (*Dicaeum cruentatum* Linnaeus) is a member of this genus found in China, India, and many Southeast Asian countries. To contribute to phylogenetic studies of *Dicaeum*, the complete mitochondrial genome of *D. cruentatum* was sequenced using a next-generation sequencing platform. The mitogenome of *D. cruentatum* (GenBank accession number MW629122) was 16,812 bp in length with an overall base composition of 30.97% A, 23.67% T, 30.28% C, and 14.07% G. The whole genome contained 13 protein-coding genes (PCGs), two ribosomal RNAs (rRNAs), and 22 transfer RNAs (tRNAs). Almost all PCGs had an ATN start codon except COX1 which started with GTG. Most of the PCGs terminated with a complete stop codon, but ND2, COX3, and ND4 had incomplete stop codons. The 16S rRNA was 1599 bp in length, while 12S rRNA was 986 bp. The *Dicaeum* species showed monophyletic clustering in the ML phylogeny and shows *D. cruentatum* as a sister taxa of *D. eximium* and *D. concolor*. This new mitogenome will provide genomic resources for future phylogenetic studies in passerines.

*Dicaeum* is a genus of passerine birds in the flowerpecker family (Dicaeidae) found throughout tropical Asia and Australasia, from India to the Philippines, and south to Australia (Cheke and Mann 2008). The genus is closely related to the genus *Prionochilus*, with which it forms a monophyletic clade (Nyári et al. 2009). The scarlet-backed flowerpecker (*Dicaeum cruentatum* Linnaeus) is a species which is found in China, India, and many Southeast Asian countries (e.g. Cambodia, Indonesia, and Malaysia) (Nyári et al. 2009). Its natural habitat is subtropical or tropical moist lowland forests (Cheke and Mann 2008). This bird actively moves about trees and shrubs, where it feeds on small fruits.

To inform phylogenetic studies of *Dicaeum* species, we sequenced the complete mitochondrial genome of *D. cruentatum* on a next-generation sequencing platform. One scarlet-backed flowerpecker bird was captured from southwestern China (E101°32′28″, N21°27′34.2″; Mengla, Yunnan Province, China) in 2020. A blood sample was taken and stored at −80 °C at the herbarium of the College of Biological Resources and Food Engineering, Qujing Normal University (voucher number QJNU-Zhu-20200805-Dc; Lizhou Tang biologytang@163.com). Genomic DNA was extracted using a blood DNA isolation kit (TIANGEN, Beijing, China). The DNA library was constructed and paired-end sequencing with a read length of 150 bp (PE150) was done on a MGI-SEQ 2000 sequencing platform by Frasergen Biotechnology Co., Ltd. (Wuhan, China). The raw data were assembled using NOVOPlasty 2.7.2 (Nicolas et al. 2017). MITOS (Bernt et al. 2013) was used to annotate of the assembled mitogenome, and annotations were adjusted manually by comparing the annotated genome with those of other *Dicaeum* species. For the phylogenetic analysis, mitogenomes of 11 another Passeriformes birds were downloaded from the NCBI nucleotide database, and the mitogenome sequences of all species were aligned with MEGA 7 (Kumar et al. 2016) using the ClustalW algorithm (Thompson et al. 2002). A maximum-likelihood (ML) phylogenetic tree was constructed by IQTREE 1.6.12 (Lam-Tung et al. 2015). According to the Bayesian information criterion, the GTR + F+R3 substitution model was ranked as the best fit model by IQTREE (Jukes and Cantor 1969; Lam-Tung et al. 2015). To assess convergence, 1000 bootstraps were calculated for the phylogenetic analysis.

The complete assembled mitogenome of *D. cruentatum* (GenBank accession number MW629122) was 16,812 bp in length with an overall base composition of 30.97% A, 23.67% T, 30.28% C, and 14.07% G. The mitogenome contained 13 protein-coding genes (PCGs), two ribosomal RNAs (rRNAs), and 22 transfer RNAs (tRNAs). Its gene content and organization were identical to those of the closely related species, *D. eximium*. Overall pairwise identity between the mitogenomes of these two species was 91%. All PCGs had an ATN start codon except COX1, which started with GTG. In addition, most of the PCGs terminated with a complete stop codon, but ND2, COX3, and ND4 had incomplete stop codons (T–– or TA–) (Table S1). The 16S rRNA was 1599 bp long with 56.0% AT content, and the 12S rRNA was 1599 bp with 50.1% AT content. The sequence length of tRNAs ranged from 66 bp to 75 bp, with an average length of 70 bp. Based on the ML phylogenetic tree, four *Dicaeum* species (*D. cruentatum*, *D. agile*, *D. concolor*, and *D. eximium*) form a monophyletic clade with high supporting values (100% bootstrap support) (Figure 1). The new mitogenome sequence of *D. eximium* will provide additional genomic resources for future phylogenetic studies of the *Dicaeum* genus.

**Figure 1.**
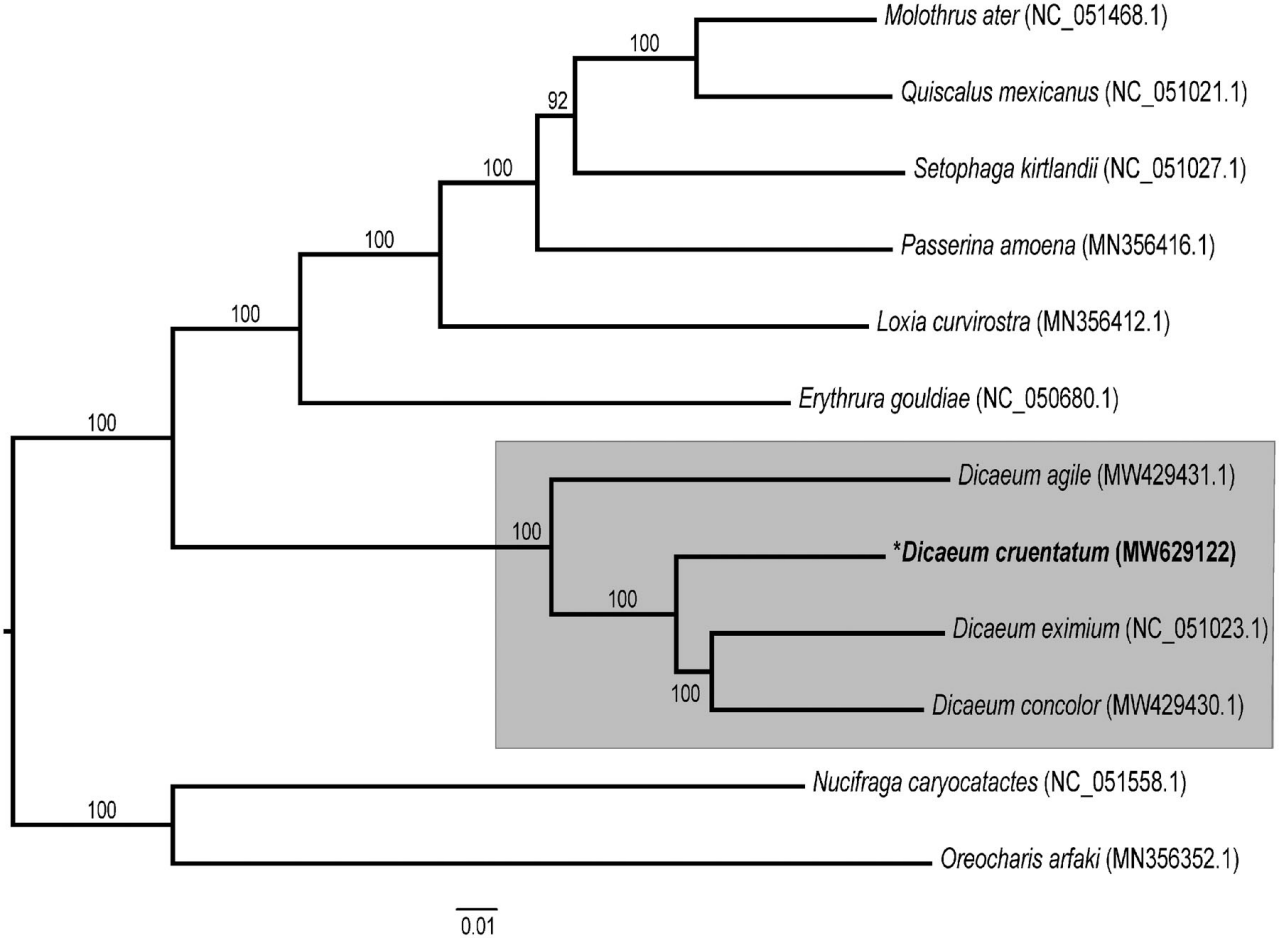
The maximum-likelihood (ML) tree constructed using mitochondrial genome sequences from *Dicaeum cruentatum* and 11 other *Passeroidea* species; *Nucifraga caryocatactes* and *Oreocharis arfaki* was used as the outgroup. Bootstrap values based on 1000 replicates are shown for each node.

## Data Availability

The genome sequence data that support the findings of this study are openly available in GenBank of NCBI at https://www.ncbi.nlm.nih.gov/ under the accession no. MW629122. The associated BioProject, SRA, and Bio-Sample numbers are PRJNA732251, SRR14630297, and SAMN19314876, respectively.
